# Comparative effectiveness of dolutegravir + lamivudine versus three‐drug regimens in Swedish clinical practice: a nationwide study

**DOI:** 10.1002/jia2.70054

**Published:** 2026-02-26

**Authors:** Erik Sörstedt, George Nduva, Fredrik Månsson, Åsa Mellgren, Johanna Repits, Eva Fernvik, Adam Stubbs, Melanie Schroeder, Johanna Brännström, Christina Carlander

**Affiliations:** ^1^ Department of Infectious Diseases Institute of Biomedicine University of Gothenburg Sahlgrenska Academy Gothenburg Sweden; ^2^ Department of Infectious Diseases Region Västra Götaland Sahlgrenska University Hospital Gothenburg Sweden; ^3^ GSK Sweden Stockholm Sweden; ^4^ Clinical Infection Medicine Department of Translational Medicine Lund University Malmö Sweden; ^5^ ViiV Healthcare London UK; ^6^ Department of Infectious Diseases/Venhälsan Stockholm Sweden; ^7^ Department of Clinical Science and Education Karolinska Institutet Stockholm Sweden; ^8^ Department of Medicine Huddinge Karolinska Institutet Stockholm Sweden; ^9^ Department of Infectious Diseases Karolinska University Hospital Stockholm Sweden

**Keywords:** antiretroviral therapy, dolutegravir, HIV‐1, lamivudine, regimen switch, two‐drug regimen

## Abstract

**Introduction:**

HIV guidelines recommend switching from a three‐drug regimen (3DR) to dolutegravir + lamivudine (DTG+3TC) for eligible individuals. This retrospective national cohort study used Swedish InfCareHIV registry data to evaluate long‐term outcomes of adults with HIV RNA <50 copies/ml who switched to DTG+3TC or a guideline‐recommended 3DR between July 2019 and May 2023 in routine clinical care.

**Methods:**

Demographic and clinical data were obtained from InfCareHIV at baseline, 6, 12, 24, 36 and 42 months post‐switch. The primary endpoint was virologic failure (VF) rates at each time point; secondary endpoints included VF rates in prespecified subgroups, time to VF, and incidence of viral blips and treatment‐emergent resistance. Generalized estimating equations modelling was used to assess the effects of clinical predictors on VF.

**Results:**

A total of 1125 individuals (46%) switched to DTG+3TC, and 1336 (54%) switched to 3DR. Adjusted VF rates post‐switch were 0.1–2.9% in the DTG+3TC group and 0.3–2.2% in the 3DR group in the intent‐to‐treat analysis (0–0.4% and 0.3–2.3% in the on‐treatment [OT] analysis, respectively). In the OT set, the odds of VF were significantly lower for DTG+3TC versus 3DR at 24, 36 and 42 months (*p*<0.001). Treatment‐emergent resistance rates were low in both groups.

**Conclusions:**

In this long‐term, real‐world, national cohort, switching to DTG+3TC was associated with low rates of VF and antiretroviral therapy resistance, indicating that eligible individuals can be switched to DTG+3TC without increased risk of VF.

## 1 INTRODUCTION

Three‐drug antiretroviral regimens (3DR) have long been the standard of care offered to people living with HIV (PLHIV). Dolutegravir (DTG), a second‐generation integrase strand transfer inhibitor (INSTI), is included in international and Swedish guidelines as a cornerstone of antiretroviral therapy (ART) in combination with emtricitabine + tenofovir or other guideline‐recommended nucleoside/nucleotide backbones such as abacavir/lamivudine, and in some cases with boosted protease inhibitors (PIs) or non‐nucleoside reverse transcriptase inhibitors (NNRTIs) where clinically indicated [1, 2, 3]. Current international and Swedish HIV treatment guidelines recommend DTG+3TC as an option for eligible individuals, but switching is not mandatory and treatment decisions are individualized, based on clinician judgement and patient preference. The main rationale for DTG+3TC is for simplification, prevention of long‐term toxicities and/or avoidance of drug–drug interactions while maintaining durable viral suppression, rather than concerns regarding triple therapy efficacy. The fixed two‐drug regimen of DTG in combination with lamivudine (3TC) was approved by the United States Food and Drug Administration and the European Medicines Agency in 2019, based on the results from phase 3 clinical trials showing durable ART efficacy together with favourable safety and tolerability in ART‐naïve individuals [4, 5, 6], and non‐inferior efficacy versus 3DR containing tenofovir has also been established [7, 8, 9, 10]. International and Swedish ART guidelines recommend switching to a two‐drug regimen with DTG+3TC in virally suppressed individuals with no history of virologic failure (VF), no hepatitis B virus (HBV) coinfection and no known or suspected resistance to 3TC or INSTIs, and in treatment‐naïve individuals [1, 2, 3]. The non‐inferiority of DTG+3TC compared to 3DR observed in clinical trials has been confirmed in routine clinical practice in Europe, Asia and America [11, 12, 13, 14, 15, 16, 17]; however, real‐world, nationwide data remains limited.

InfCareHIV is a national quality registry and decision support tool for clinical HIV care in Sweden, and comprises clinical, virological and epidemiological data for more than 99% of all diagnosed PLHIV in Sweden, thus providing an almost complete picture of Swedish clinical practice [18]. In this paper, we report findings from a comparative cohort study based on retrospective InfCareHIV data. This study used retrospective InfCareHIV data to evaluate the clinical effectiveness of DTG+3TC versus guideline‐recommended 3DRs in virologically suppressed, treatment‐experienced PLHIV in routine clinical care in Sweden.

## 2 METHODS

### 2.1 Study design

This was a national, retrospective, observational, comparative cohort study conducted between July 2019 and May 2023, comprising adult PLHIV included in InfCareHIV, with undetectable viral load (VL; HIV RNA <50 copies/ml), and switched to either DTG+3TC or a 3DR included in the Swedish ART treatment guidelines during the study period. Individuals could have switched from any guideline‐approved 3DR either to DTG+3TC or to another 3DR. This reflects real‐world clinical practice where a switch to DTG+3TC represents proactive regimen simplification, whereas a switch to another 3DR may be motivated by side effects, adherence considerations or other clinical factors. No minimum duration of viral suppression was required. Individuals with hepatitis B coinfection or documented prior VF/resistance to INSTI or 3TC were not considered eligible for DTG+3TC. The presence of comorbidities did not constitute an exclusion criterion for switching to DTG+3TC. Demographic and clinical data (including patient‐reported outcome measures [PROMs] where available) were obtained from InfCareHIV.

The study was conducted in accordance with the ethical principles outlined in the Declaration of Helsinki, and consistent with the International Council for Harmonisation of Technical Requirements for Pharmaceuticals for Human Use guidelines on Good Clinical Practice and applicable regulatory requirements. Ethical approval was obtained from the Swedish Ethical Review Authority (application ref 2022‐05624‐01).

### 2.2 Data source

Established in 2003, InfCareHIV currently includes all 29 clinical HIV centres in Sweden and covers more than 99% of all diagnosed PLHIV living in Sweden.

At the time of diagnosis, all individuals are informed about the InfCareHIV registry, their right to opt out and that their de‐identified data may be used for research purposes. For this study, ethical approval was obtained (Dnr 2022‐05624‐01), including a waiver of individual consent due to the use of de‐identified data and the minimal risk involved. Data collected at enrolment include socio‐demographic data (including age, sex at birth, gender identity, country of birth) and HIV characteristics (date of any last negative and first positive HIV test, mode and suspected country of HIV acquisition, confirmed primary HIV acquisition, HBV and hepatitis C (HCV) serostatus). Laboratory and ART data are collected and updated at each follow‐up visit, including ART start and stop dates, doses and mode of ART administration, VL and CD4 cell count, confirmed resistance mutations and reasons for change [18]. Treatment‐emergent resistance was defined as the occurrence of new resistance‐associated mutations (RAMs) detected on genotypic testing after VF and not present at baseline.

In 2011, a nine‐item self‐reported health questionnaire was integrated into the InfCareHIV registry to collect data for systematic quantification of PROMs [19, 20]. An English version of the questionnaire is provided in Table S1. The aim is for all participants to be invited to complete the questionnaire annually, either online or during an outpatient clinic visit. The questionnaire is available in several languages and captures PROMs relating to physical, mental and sexual health, smoking habits, self‐reported ART adherence, defined as any missed dose of ART within the preceding week, experience of side effects, and patient‐reported experience measures of involvement and satisfaction with care. The responses are used to guide follow‐up consultations and enhance person‐centred HIV care [18, 19]. The integration of the health questionnaire into InfCareHIV offers a holistic view of each individual's health and wellbeing and facilitates person‐centred HIV care, focusing the consultation on the individual's current needs [19].

### 2.3 Endpoints and outcome assessments

Outcomes were assessed at prespecified follow‐up time points (6, 12, 24, 36 and 42 months). VLs measured closest to these time points were used without applying an additional window. The primary endpoint was the proportion of study participants with VF at 6, 12, 24, 36 and 42 months post‐switch. VF was defined as either two consecutive HIV RNA ≥200 copies/ml, or one HIV RNA ≥200 copies/ml followed by discontinuation of the core agent. Secondary endpoints included post‐switch time to VF, incidence of viral blips (defined as having a single measurement of HIV RNA ≥50–200 copies/ml after suppression to <50 copies/ml at any time point post‐switch) and treatment‐emergent ART resistance, and changes in self‐reported health status, wellbeing and ART adherence as captured in the InfCareHIV health questionnaire.

The study protocol also specified a range of exploratory *post‐hoc* analyses of potential predictors of virologic outcomes in the DTG+3TC and 3DR group, respectively.

### 2.4 Statistical analysis

The study baseline was defined as the time point for switching treatment to either DTG+3TC or a guideline‐approved 3DR regimen. The time on study treatment was defined as the difference between the date of switching to study treatment and either the study end date, the date of study treatment discontinuation, loss to follow‐up or death, whichever happened first.

Two complementary analytic sets were used. In the intent‐to‐treat (ITT) analysis, all participants with available laboratory data at a given time point were included, irrespective of treatment continuation. In the on‐treatment (OT) analysis, participants were required to have both available laboratory data and to be on ongoing study treatment at the given time point; discontinuation, therefore, led to censoring in the OT but not the ITT analysis. These definitions were intended to provide complementary perspectives on regimen effectiveness in routine care, recognizing that they differ from strict randomized controlled trials‐based ITT/per‐protocol definitions. The proportion of study participants with VF at each time point was estimated as the number of study participants with VF divided by the number of study participants with available laboratory measurements at that time point (ITT analysis set), or as the number of study participants with VF divided by the number of study participants with available laboratory measurements and ongoing study treatment (the OT analysis set).

For modelling, a logistic generalized estimating equations (GEE) model was applied to account for repeated measures within individuals and to accommodate missing data patterns common in registry data. Exploratory analyses of associations between covariates and VF were performed to provide additional insight into patient and treatment characteristics influencing outcomes. The following covariates were included in the GEE model: treatment group (DTG+3TC vs. 3DR), post‐switch ART adherence, sex at birth, age, baseline CD4 count, mode of HIV acquisition, HIV subtype, baseline drug resistance mutations, HBV and HCV serostatus, and viral blips. To evaluate the treatment effect at each specified time point, linear contrasts were created that assessed differences between ART groups at each time point. Significance testing for linear contrasts was performed to determine the statistical significance of the treatment effect at each time point, with correction for multiple testing using a single‐step method. Adjusted odds ratios (ORs) with 95% confidence intervals (CIs) from the models were reported with corresponding *p*‐values. Two‐sided *p*‐values <0.05 were considered statistically significant. The incidence of treatment‐emergent resistance was calculated as the total number of events divided by the total number of years under study contributed by all study participants. The prevalence of known drug resistance was calculated as the total number of study participants with any known RAMs divided by the total number of study participants. The incidence and prevalence estimates were reported with corresponding 95% CIs. The missing indicator method was used to study patterns associated with missing data for HIV subtype, post‐switch ART adherence and mode of HIV acquisition.

## 3 RESULTS

### 3.1 Study population

**Figure 1:**
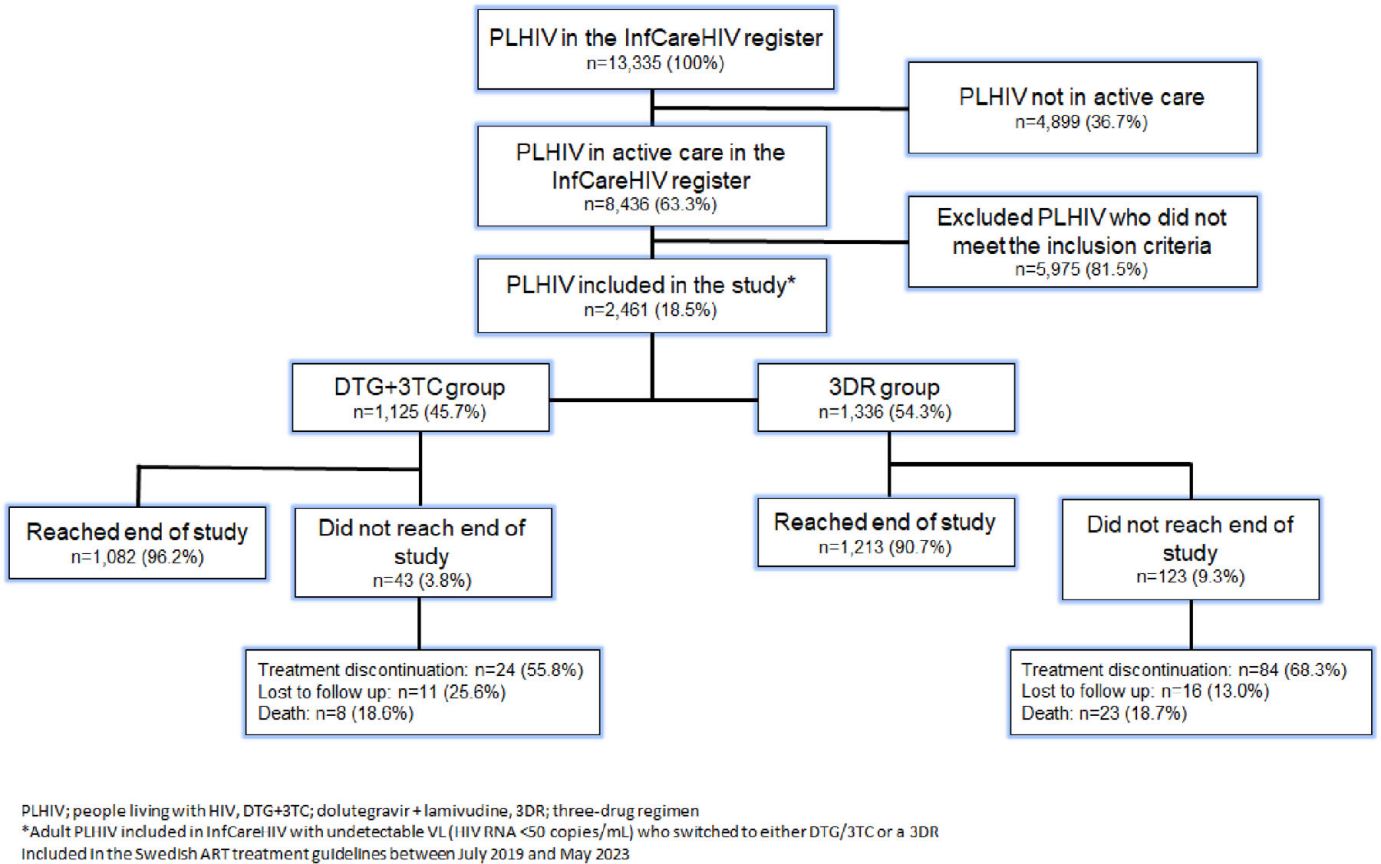
Flow chart of the study population.

At the time of the retrospective analysis, the InfCareHIV registry contained the records of a total of 8436 PLHIV receiving active HIV care (Figure 1). Of these, 2461 (29.2%) individuals fulfilled the study eligibility criteria of having undetectable VL (HIV RNA <50 copies/ml) and having switched to either DTG+3TC or a 3DR included in the Swedish ART treatment guidelines between 3 July 2019 and 29 May 2023. Individuals included in this analysis were diagnosed with HIV between 1980 and 2022. The most common pre‐switch regimens were bictegravir + emtricitabine + tenofovir alafenamide, DTG + emtricitabine + tenofovir disoproxil fumarate, DTG + 3TC + abacavir and DTG + emtricitabine + tenofovir alafenamide. Reasons for switching were not systematically recorded in the registry; however, in clinical practice, switches to DTG+3TC typically reflected regimen simplification in stable patients, whereas switches to other 3DRs were more often motivated by side effects, adherence considerations or other clinical factors.

A total of 1125 eligible participants (46%) switched to DTG+3TC, and 1336 (54%) switched to another guideline‐recommended 3DR, where the most common combinations were bictegravir + emtricitabine + tenofovir alafenamide (BIC+FTC+TAF, *n* = 544, 40.7%), DTG+ emtricitabine + tenofovir disoproxil fumarate (DTG+FTC+TDF, *n* = 296, 22.2%), DTG+3TC+ abacavir (DTG+3TC+ABC, *n* = 210, 15.7%) and DTG+FTC+TAF (*n* = 130, 9.7%).

Individuals switching to DTG+3TC were significantly more likely to be older (mean age [SD] at baseline 50.1 [13.0] years) than those switching to a guideline‐approved 3DR (47.5 [15.0] years), and also more likely to be from Europe or North America (577 individuals [51%] in the group switching to DTG+3TC vs. 488 individuals [37%] in the 3DR group), born male (737 [66%] vs. 723 [54%] individuals) and have CD4 cell counts ≥500 cells/ml (696 [62%] vs. 761 [57%] individuals) at study baseline (*p*<0.0001 for all). In addition, significantly fewer study participants switching to DTG+3TC had pre‐switch viral blips (391 individuals [35%], vs. 587 individuals [44%] switching to 3DR; *p*<0.0001) and/or known *M184V* RAMs (10 individuals [0.9%] vs. 86 individuals [6.4%] switching to 3DR; *p* = 0.006, Table 1).

**Table 1:** Baseline characteristics of PLHIV included in InfCareHIV who switched to either DTG+3TC or a three‐drug ART regimen between July 2019 and May 2023

Characteristic	DTG+3TC (*n* = 1125)	3DR (*n* = 1336)	Total (*n* = 2461)	*p* value
Age at baseline (years), mean (SD)	50.1 (13.0)	47.5 (15.0)	48.7 (14.0)	<0.0001
Age at diagnosis (years), mean (SD)	36.1 (12.0)	33.0 (13.0)	34.4 (13.0)	<0.0001
Sex at birth, *n* (%)				<0.0001
Male	737 (66)	723 (54)	1460 (59)	
Female	387 (34)	613 (46)	1000 (41)	
Missing	1 (0.09)	0 (0.0)	1 (0.04)	
Time on ART, years (mean, SD)	12.8 (6.9)	13.7 (7.8)	13.3 (7.4)	0.001
Baseline VL (copies/ml), median (range)	0 (0–49)	0 (0–49)	0 (0–49)	
Mode of acquisition, *n* (%)				<0.0001
Heterosexual	541 (48)	729 (55)	1270 (52)	
MSM	436 (39)	376 (28)	812 (33)	
PWID	34 (3)	59 (4.4)	93 (3.8)	
Perinatal	11 (1.0)	77 (5.8)	88 (3.6)	
Other/missing	103 (9.2)	95 (7.1)	198 (8.0)	
Geographical origin, *n* (%)				<0.0001
Europe and North America	577 (51)	488 (37)	1056 (43)	
Sub‐Saharan Africa	267 (24)	520 (39)	787 (32)	
Asia and the Pacific	140 (12)	151 (11)	291 (12)	
Other/missing	150 (13)	177 (13)	318 (13)	
CD4 count at baseline, *n* (%)				<0.0001
<500	248 (26)	422 (36)	670 (31)	
≥500	696 (74)	761 (64)	1457 (69)	
Missing	181 (16)	153 (11)	334 (14)	
^a^Pre‐switch adherence, *n* (%)				0.404
Optimal	815 (72)	832 (62)	1647 (67)	
Sub‐optimal	56 (5.0)	68 (5.1)	124 (5.0)	
Missing	254 (23)	436 (33)	690 (28)	
^b^Pre‐switch low‐level viraemia, *n* (%)				<0.0001
No	734 (65)	749 (56)	1483 (60)	
Yes	391 (35)	587 (44)	978 (40)	
Pre‐switch *M184V* RAMs^c^, *n* (%)	10 (0.9)	86 (6.4)	96 (3.9)	0.006

Abbreviations: 3DR, three‐drug regimen; ART, antiretroviral therapy; DTG+3TC, dolutegravir + lamivudine; MSM, men having sex with men; PWID, people who inject drugs; RAM, resistance‐associated mutation.

^a^ Patients were classified as having sub‐optimal adherence if they reported not having taken any HIV medication or if they had missed any doses during the week prior to completing the HRQOL questionnaire. Otherwise, their adherence was classified as optimal. Pre‐switch suboptimal adherence was 5.0% and 5.1% in the DTG+3TC and 3DR groups, respectively.

^b^ Low‐level viraemia was defined as the incidence of HIV‐1 RNA measures of >50 copies/ml and <200 copies/ml after suppression to <50 copies/ml at any time point. Pre‐switch incidence of low‐level viraemia was 35% and 44% in the DTG+3TC and 3DR groups, respectively.

^c^ The most common pre‐switch RAMs in the DTG+3TC group were: *M36I* (*n* = 202, 18%), *L89M* (*n* = 181, 16%), *I93L* (*n* = 155, 14%), *L63P* (*n* = 102, 9%) and *H69K* (*n* = 89, 8%). The most common pre‐switch RAMs in the 3DR group were: *M36I* (*n* = 229, 17%), *L89M* (*n* = 215, 16%), *I93L* (*n* = 175, 13%), *L63P* (*n* = 148, 11%) and *H69K* (*n* = 110, 8%).

Table 2 shows the size of the study population at each time point in the ITT and OT analysis sets.

**Table 2:** Sample sizes (*n*) at each time point in the ITT and OT analysis sets

		6 months		12 months		24 months		36 months		42 months	
	Baseline	ITT	OT	ITT	OT	ITT	OT	ITT	OT	ITT	OT
DTG+3TC	1125	773	773	558	551	308	304	112	107	35	32
3DR	1336	1005	1005	860	835	677	644	332	312	139	132
Total	2461	1778	1778	1418	1386	985	948	444	419	174	164

Abbreviations: DTG+3TC, dolutegravir + lamivudine; ITT, intent‐to‐treat; OT, on‐treatment; 3DR, three‐drug regimen.

Overall, the ART regimens in the study were well tolerated, with only seven participants (0.6%) in the DTG+3TC group and 11 (0.8%) in the 3DR group discontinuing treatment due to side effects.

### 3.2 Post‐switch VF counts and rates (primary endpoint)

The proportion of study participants with VF was generally low in both ART groups at each time point (Table 3). During the 42‐month study period, the VF rates per 10,000 study participants on DTG+3TC and 3DR ranged from 0.1% to 2.9% and 0.3% to 2.2% in the ITT set and from 0% to 0.4% and 0.3% to 2.3% in the OT set, respectively (Table 3A). Likewise, the absolute number of VF events during the study was lower in the DTG+3TC group compared with the 3DR group at all time points in both analysis sets (Table 3B). In the ITT set, a total of seven participants in the DTG+3TC group and 37 in the 3DR group fulfilled the criteria for VF during the study. Similar results were noted in the OT set, with VF counts of three participants in the DTG+3TC group and 28 in the 3DR group, respectively.

**Table 3:** Proportion of study participants with VF at each time point (primary endpoint) in the ITT and OT analysis sets

ART group	*N* (baseline)	6 months (*n* = 1778)	12 months (*n* = 1418)	24 months (*n* = 985)	36 months (*n* = 444)	42 months (*n* = 174)
**A. VF rates per 10,000 individuals (95% CI)**						
*ITT set*						
DTG+3TC	1125	12.9 (1.8, 91.5)	35.8 (9.0, 143)	32.5 (4.6, 230)	179 (45.3, 707)	286 (41.4, 1970)
3DR	1336	29.9 (9.7, 92.6)	140 (79.8, 246)	177 (101, 310)	211 (101, 439)	216 (70.5, 662)
Total	2461	22.5 (8.5, 59.9)	98.7 (58.6, 166)	132 (76.9, 227)	203 (106, 388)	230 (87.3, 606)
*OT set*						
DTG+3TC	1125	12.9 (1.8, 91.5)	36.3 (9.1, 145)	0.0 (0.0, 0.0)	0.0 (0.0, 0.0)	0.0 (0.0, 0.0)
3DR	1336	29.9 (9.7, 92.6)	108 (56.4, 207)	109 (52.2, 228)	192 (86.9, 424)	227 (74.2, 695)
Total	2461	22.5 (8.5, 59.9)	79.4 (44.1, 143)	73.8 (35.3, 154)	143 (64.6, 316)	183 (59.6, 562)
**B. Absolute VF counts (*n*)**						
*ITT set*						
DTG+3TC	1125	1	2	1	2	1
3DR	1336	3	12	12	7	3
Total	2461	4	14	13	9	4
*OT set*						
DTG+3TC	1125	1	2	0	0	0
3DR	1336	3	9	7	6	3
Total	2461	4	11	7	6	3

*Note*: Within‐group estimates of precision for VF rates following switch to DTG+3TC or 3DR, with 95% CI.

Baseline study populations: DTG+3TC group (*n* = 1125), 3DR group (*n* = 1336), total study group (*n* = 2461). Overall, in the ITT analysis set, seven patients on DTG+3TC and 37 patients on 3DR had VF; in the OT analysis set, three patients on DTG+3TC and 28 patients on 3DR had VF.

Abbreviations: ART, antiretroviral therapy; CI, confidence interval; DTG+3TC, dolutegravir + lamivudine; ITT, intent‐to‐treat; OT, on‐treatment; VF, virologic failure; 3DR, three‐drug regimen.

*Post hoc* modelling of potential treatment effects showed no difference between the ART groups with respect to the odds of VF at any time point in the ITT analysis set. In the OT set, the odds of having VF were significantly lower on DTG+3TC compared with 3DR at 24, 36 and 42 months (*p*<0.001 for all). Additional sensitivity analyses confirmed these results. In both the ITT and OT analysis sets, suboptimal adherence and viral blips post‐switch, together with documented ART resistance at baseline and post‐switch, were associated with higher odds of VF, while older age decreased the odds. The complete results of the *post hoc* analyses of ITT and OT predictors of VF are shown in Tables S2 and S3.

### 3.3 Time to VF

**Figure 2:**
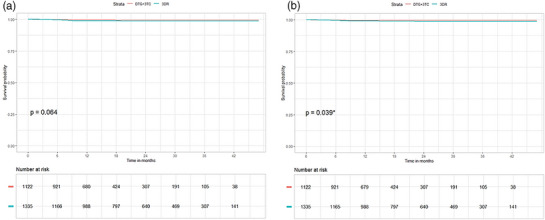
Time to VF in the ITT (a) and OT (b) analysis sets.

Figure 2 shows the estimated probabilities of VF‐free survival at each post‐switch time point in the ITT and OT analysis sets, respectively. No significant difference in the time to VF was seen between the DTG+3TC and 3DR groups in the ITT analysis; a significant difference that was observed in the OT set disappeared when the analysis was adjusted for confounding variables. Older age and male sex were significant predictors of longer time to VF in both the ITT and OT analyses, whereas missing RAM information at baseline was predictive of shorter time to VF. Baseline CD4 count <500 was also a significant predictor of longer time to VF in the ITT, but not the OT analysis. The full results of the proportional hazards testing for the time to VF are shown in Tables S4 and S5.

### 3.4 Incidence of post‐switch viral blips

The overall incidence of post‐switch viral blips was significantly lower among study participants who switched to DTG+3TC versus 3DR (3.01 vs. 5.38 per 100 person‐years [PY], *p*<0.001). Having a history of low‐level viraemia and/or a baseline CD4 count ≥500 both increased the odds of viral blips during the study. The odds of having viral blips were significantly lower in the DTG+3TC group versus 3DR at 6 and 42 months in both the ITT (*p* = 0.02 and *p*<0.001, respectively) and OT analysis sets (*p* = 0.01 and *p*<0.001, respectively).

### 3.5 Incidence of treatment‐emergent ART resistance

The incidence of treatment‐emergent resistance post‐switch was low in the study population. In the DTG+3TC group, the unmasked prevalence of NNRTI resistance was 0.123 (95% CI 0.031–0.491) per 100 PY, and the incidence of nucleoside/nucleotide reverse transcriptase inhibitor (NRTI) resistance was 0.061 (95% CI 0.009–0.436) per 100 PY. The NRTI mutations that arose in the DTG+3TC group after the switch were *M184V*, *K65R*, *A62V*, *S68G*, *K219R* and *K219E*. No case of PI resistance was observed in the DTG+3TC group. Conversely, in the 3DR group, there was no case of NNRTI resistance, while the incidences of NRTI and PI resistance were 0.531 (0.315–0.895) and 0.114 (0.037–0.352) per 100 PY, respectively. No treatment‐emergent INSTI resistance was observed post‐switch in either group.

### 3.6 Post‐switch health questionnaire responses

A total of 893 study participants (36%) had completed at least one health questionnaire within 1 year prior to switching, and 651 (26%) had completed a questionnaire within 1 year after switching. Both pre‐ and post‐switch questionnaires were available for 375 participants (15%). Approximately two‐thirds of the study participants (69% in the DTG+3TC group and 59% in the 3DR group) reported being “very satisfied” or “satisfied” with their physical health before switching. After switching, the proportion of study participants who were “very satisfied” or “satisfied” with their physical health was 62% in the DTG+3TC group and 55% in the 3DR group. Similar results were reported with respect to the proportions of study participants being “very satisfied” or “satisfied” with their mental health (65% and 63% pre‐switch; 62 and 66% post‐switch in the DTG+3TC and 3DR groups, respectively) and with their sex life (55% and 43% pre‐switch; 52 and 42% post‐switch). The vast majority of the study participants who completed the questionnaires were “very satisfied” or “satisfied” with the HIV care provided at their outpatient clinic (95% and 93% pre‐switch; 97 and 94% post‐switch).

Self‐reported treatment adherence was very high in the questionnaires, both pre‐ and post‐switch. Before switching, 88% of the study participants who completed the questionnaire reported not missing a single dose of medication in the previous week; after switching, this level of adherence was reported by 93% in the DTG+3TC and 87% in the 3DR group. Only two participants in the 3DR group reported missing three or more doses in the previous week after switching.

Among the study participants who completed the questionnaire, 12% reported experiencing side effects prior to switching, and 10% reported being “very bothered” or “bothered” by side effects. After switching, the proportion of study participants experiencing side effects dropped to 8% in the DTG+3TC and 11% in the 3DR group, and 5% and 8%, respectively, remained “very bothered” or “bothered” by side effects post‐switch. The full results of the pre‐ and post‐switch health questionnaires are shown in Table S6.

## 4 DISCUSSION

This study offers nationwide real‐world evidence from Sweden, demonstrating high virologic effectiveness, low risk of viral blips or resistance, and high levels of self‐reported health status and adherence in PLHIV switching to DTG+3TC. These results are consistent with results from randomized controlled trials [7, 8, 9, 10] and previously reported real‐world studies [11, 12, 13, 14, 15, 16, 17], and support switching to DTG+3TC in stable, virologically suppressed, treatment‐experienced PLHIV in routine clinical care. As PLHIV live longer, with co‐morbidities and polypharmacy, 2DR provide an option to potentially minimize long‐term toxicities and reduce drug−drug interactions while maintaining effective viral suppression. Switching to DTG+3TC eliminates the risk of interactions with booster drugs such as ritonavir and cobicistat, and reduces the risks associated with specific treatments such as decreased bone mineral density and nephrotoxicity linked to tenofovir [1, 21, 22] and the potentially increased risk of cardiovascular events with abacavir, as well as NNRTI‐related hepatotoxicity [23].

Multiple real‐world studies have confirmed that DTG‐based 2DR are effective and well‐tolerated in routine clinical practice in Europe and the United States [24, 25, 26]—and across a range of populations [27]. Previous real‐world studies, including a US study of 787 individuals switching to DTG+3TC, showed high virologic effectiveness, with low‐level viraemia in 118 cases in 14 months of follow‐up, with low rates of VF [11]. Likewise, a retrospective, observational study of 324 individuals in an Italian treatment centre showed that 99% of participants who switched to DTG+3TC achieved undetectable VL over a period of up to 30 months, compared with 97% of those who remained on a 3DR [12]. Similar durable virologic effectiveness has been reported over periods of up to 144 weeks in cohorts from Italy [13] and Spain [15]. A systematic review of real‐world studies on DTG+3TC, involving over 5000 PLHIV, also reported virologic suppression rates of 97–100% and VF rates of 0–3.3 per 100 PY [16].

A strength of this study is its nationwide coverage, and a large cohort of over 2400 individuals switching therapy, with nearly 4 years of follow‐up. Nevertheless, the number of participants with available data decreased over time, with fewer individuals contributing at the later follow‐up points. In particular, the 42‐month estimates are based on a small proportion of the baseline population, reflecting shorter follow‐up for those who switched later in the study period. This attrition limits precision and may bias longer‐term estimates; however, including all eligible individuals maximized representativeness of real‐world practice, and outcomes are reported separately for each time point to allow transparent interpretation. PROM results were limited by under‐reporting, yet the majority of study participants with pre‐ and post‐switch data were satisfied with their physical, mental and sexual health. The observation that satisfaction rates appeared to decrease slightly post‐switch may be explained by non‐ART‐related factors, such as the COVID‐19 pandemic or a general age‐related decline in physical health during the study period.

Interpreting the results requires consideration of the inherent differences between the DTG+3TC and 3DR groups, driven by distinct selection criteria. Clinicians tailored switch decisions based on clinical factors and patient preferences, with DTG+3TC typically chosen for individuals with strong adherence, fewer treatment failures and minimal resistance, while 3DR switches were often prompted by side effects or adherence issues. These differences preclude direct statistical comparisons, such as propensity score matching. Differences in baseline characteristics between the DTG+3TC and 3DR groups, including higher CD4 counts, fewer viral blips and lower prevalence of resistance mutations in the DTG+3TC group, may partly explain the lower rates of VF observed. Nevertheless, this study effectively illustrates current clinical practice for treatment selection in Sweden, providing valuable real‐world insights into the use and outcomes of these regimens in specific patient populations.

## 5 CONCLUSIONS

This long‐term real‐world study included 2461 PLHIV who switched to either DTG+3TC or 3DR during a 4‐year period and showed low rates of VF in both treatment groups. Clinical and demographic factors, such as older age and higher baseline CD4 count, which may both be proxies for adherence to ART, were associated with decreased odds of VF, while low‐level viraemia, suboptimal adherence and drug resistance increased the odds. Overall, our study supports the effectiveness of DTG+3TC as a treatment option in routine clinical practice, confirming its high barrier to resistance and low rates of VF, consistent with findings from randomized controlled trials.

## COMPETING INTERESTS

ES has received lecture fees from Gilead Sciences, FM has received honoraria for advisory boards from GSK/ViiV Healthcare and lecture fees paid to his department from Gilead Sciences and from AstraZeneca. ÅM has received lecture fees from Gilead Sciences and GSK/ViiV Healthcare and honoraria for Advisory Boards from GSK/ViiV. JB has received honoraria for Advisory Boards from GSK/ViiV Healthcare, Gilead Sciences and MSD. JB has also received an unrestricted research grant from Gilead Sciences. CC has received lecture and consulting fees from GSK/ViiV Healthcare, MSD and Gilead Sciences paid to her department. CC has also received an unrestricted research grant from Gilead Sciences. GN and JR are employees of GSK Sweden; EF, AS and MS are employees of ViiV Healthcare.

## AUTHOR CONTRIBUTIONS

ES, FM, ÅM, JB and CC contributed to study design, data collection and data analysis or interpretation. GN, SH, JR, EF, AS and MS contributed to study design and data analysis or interpretation. All authors have read and approved the text as submitted.

## FUNDING

Funding for statistical analysis and medical writing was provided by ViiV Healthcare. The academic authors contributed independently and received no financial compensation for their work on study design, data interpretation, or manuscript preparation.

## Supporting information




**Table S1**. The InfCareHIV Health Questionnaire, English translation.
**Table S2**. Adjusted ORs, 95% CIs and corresponding *p*‐values from GEE modelling of associations between clinical and demographic variables and VF; ITT analysis.
**Table S3**. Adjusted ORs, 95% CIs and corresponding *p*‐values from GEE modelling of associations between clinical and demographic variables and VF; OT analysis.
**Table S4**. Adjusted ORs, 95% CIs and corresponding *p*‐values from GEE modelling of time to VF; ITT analysis.
**Table S5**. Adjusted ORs, 95% CIs and corresponding *p*‐values from GEE modelling of time to VF; OT analysis.
**Table S6**. PLHIV health and treatment satisfaction rates (A), self‐reported adherence (B) and experience of side effects (C) in the InfCareHIV health questionnaire.

## Data Availability

The data that support the findings of this study are available from the corresponding author upon reasonable request.
