# Supplementary material for: Comparative effectiveness of dolutegravir + lamivudine versus three‐drug regimens in Swedish clinical practice: a nationwide study
Source: J Int AIDS Soc. 2026 Feb 26;29(3):e70054. doi: 10.1002/jia2.70054 (PMC12946511; doi:10.1002/jia2.70054)
Supplement: Supplementary file 1 — Table S1. The InfCareHIV Health Questionnaire, English translation. Table S2. Adjusted ORs, 95% CIs and corresponding p‐values from GEE modelling of associations between clinical and demographic variables and VF; ITT analysis. Table S3. Adjusted ORs, 95% CIs and corresponding p‐values from GEE modelling of associations between clinical and demographic variables and VF; OT analysis. Table S4. Adjusted ORs, 95% CIs and corresponding p‐values from GEE modelling of time to VF; ITT analysis. Table S5. Adjusted ORs, 95% CIs and corresponding p‐values from GEE modelling of time to VF; OT analysis. Table S6. PLHIV health and treatment satisfaction rates (A), self‐reported adherence (B) and experience of side effects (C) in the InfCareHIV health questionnaire. [file JIA2-29-e70054-s001.pdf]

Comparative Effectiveness of Dolutegravir + Lamivudine Versus Three-Drug Regimens in Swedish Clinical Practice: A Nationwide Study

Supplementary information

For Review Only

Table S1. The InfCareHIV Health Questionnaire, English translation.

Please tick the appropriate box for each statement

1. How satisfied are you with your physical health?

Very unsatisfied	Unsatisfied	Rather unsatisfied	Rather satisfied	Satisfied	Very satisfied
☐	☐	☐	☐	☐	☐

2. How satisfied are you with your psychological wellbeing?

Very unsatisfied	Unsatisfied	Rather unsatisfied	Rather satisfied	Satisfied	Very satisfied
☐	☐	☐	☐	☐	☐

For Review Only

3. How satisfied are you with your sexual life (regardless if you have sex with a partner or on your own)?

Very unsatisfied	Unsatisfied	Rather unsatisfied	Rather satisfied	Satisfied	Very satisfied
☐	☐	☐	☐	☐	☐

4a. Are you currently taking HIV medication?

Yes ☐ ⟶ Go to question 4b. No ☐ ⟶ Go to question 5.

4b. Do you experience any side effects?

Yes ☐ ⟶ Go to question 4c. No ☐ ⟶ Go to question 4d.

4c. To what extent are you troubled by medical side effects?

Very troubled	Troubled	Rather troubled	Not very troubled	Not at all troubled
☐	☐	☐	☐	☐

4d. How many doses have you missed the last week?

☐ 0 ☐1-2 ☐ 3 or more doses

5: Do you feel involved in the planning and realization of your HIV care and treatment?

Never	Seldom	Sometimes	Always
☐	☐	☐	☐

6. How satisfied are you with the quality of care provided at your HIV clinic?

Very unsatisfied	Unsatisfied	Rather unsatisfied	Rather satisfied	Satisfied	Very satisfied
☐	☐	☐	☐	☐	☐

Thank you for your participation!

For Review Only

Table S2. Adjusted ORs, 95% CIs and corresponding p-values from GEE modelling of associations between clinical and demographic variables and VF; ITT analysis.

**Variable**	**OR**	**2.5%**	**97.5%**	**p-value[a]**
ART group				
DTG/3TC	REF			
3DR	1.053	0.11	10.35	0.96
Intercept	0.02	0.0016	0.29	**0.0039**
Sex				
Female	REF			
Male	0.52	0.21	1.28	0.16
Age at baseline (years)	0.96	0.93	0.99	**0.0052**
Baseline CD4 count (cells/mL)				
<500	REF			
≥500	0.27	0.12	0.58	**0.00089**
Missing	0.64	0.23	1.80	0.40
Pre-existing RAMs				
No pre-existing RAMs	REF			
Pre-existing RAMs	0.99	0.51	1.95	0.99
Missing RAM information	19.87	2.98	132.37	**0.0020**
Mode of transmission				
Heterosexual	REF			
Homo/bisexual	0.39	0.11	1.43	0.16
Other	0.65	0.15	2.77	0.56
Unknown/missing	0.28	0.04	2.18	0.22
HIV-1 subtype group				
HIV-1 A	REF			
HIV-1 B	1.40	0.31	6.27	0.66
HIV-1 C	0.31	0.07	1.39	0.13
HIV-1 CRF	0.39	0.10	1.49	0.17
HIV-1 D	<0.0001	<0.0001	<0.0001	**0**
Missing	0.35	0.11	1.086	0.069
Other/Unknwon	3.11	0.67	14.42	0.15
Study adherence				
Optimal	REF			
Suboptimal	3.87	1.12	13.40	**0.033**

For Review Only

Missing	4.37	2.36	8.084	**<0.0001**
Low-level viraemia post-switch				
No	REF			
Yes	4.84	2.39	9.80	**<0.0001**
Low-level viraemia pre-switch				
No	REF			
Yes	0.94	0.46	1.93	0.87
Treatment-emergent resistance				
No	REF			
Yes	21.79	4.32	109.86	**0.00019**
Known resistance pre-switch				
No	REF			
Yes	<0.0001	<0.0001	<0.0001	**0**
HBV serostatus				
Negative	REF			
Positive	<0.0001	<0.0001	<0.0001	**0**
HCV serostatus				
Negative	REF			
Positive	<0.0001	<0.0001	<0.0001	**0**
Time				
M6	REF			
M12	3.061	0.27	34.30	0.36
M24	2.98	0.17	51.31	0.45
M36	14.74	1.074	202.40	**0.044**
M42	34.58	1.40	852.89	**0.030**
3DR*time				
3DR *M6	REF			
3DR *M12	1.92	0.12	29.74	0.64
3DR *M24	2.59	0.11	60.57	0.55
3DR *M36	0.58	0.030	11.13	0.72
3DR *M42	0.25	0.01	8.95	0.45

Abbreviations: OR; odds ratio, CI; confidence interval, GEE; generalised estimating equations, VF; virologic failure, ITT; intent to treat, RAM; resistance-associated mutation, CRF; circulating recombinant forms, ART; antiretroviral therapy, DTG/3TC; dolutegravir/lamivudine, 3DR; three-drug regimen, M; month.

[a] p values in bold are considered statistically significant.

For Review Only

Table S3. Adjusted ORs, 95% CIs and corresponding p-values from GEE modelling of associations between clinical and demographic variables and VF; OT analysis.

**Variable**	**OR**	**2.5 %**	**97.5 %**	**p-value**[a]
ART group				
DTG/3TC	REF			
3DR	0.92	0.089	9.52	0.94
Intercept	0.13	0.011	1.61	0.11
Sex				
Female	REF			
Male	0.73	0.26	2.064	0.55
Age at baseline (years)	0.94	0.90	0.97	**0.00045**
Baseline CD4 count (cells/mL)				
<500	REF			
≥500	0.40	0.16	1.018	0.055
Missing	0.32	0.048	2.21	0.25
Pre-existing RAMs				
No pre-existing RAMs	REF			
Pre-existing RAMs	0.48	0.20	1.17	0.11
Missing RAM information	14.90	2.088	106.28	**0.0071**
Mode of transmission				
Heterosexual	REF			
Homo/bisexual	0.061	0.0073	0.51	**0.010**
Other	0.24	0.042	1.38	0.11
Unknown/missing	0.38	0.046	3.040	0.36
HIV-1 subtype group				
HIV-1 A	REF			
HIV-1 B	1.27	0.24	6.71	0.78
HIV-1 C	0.22	0.054	0.91	**0.037**
HIV-1 CRF	0.29	0.063	1.34	0.11
HIV-1 D	<0.0001	<0.0001	<0.0001	**0**
Missing	0.18	0.061	0.52	**0.0016**
Other/Unknwon	0.51	0.030	8.58	0.64
Study adherence				
Optimal	REF			
Suboptimal	5.021	1.29	19.47	**0.020**

For Review Only

Missing	3.88	2.00	7.50	**<0.0001**
Low-level viraemia post-switch				
No	REF			
Yes	8.45	3.70	19.31	**<0.0001**
Low-level viraemia pre-switch				
No	REF			
Yes	1.030	0.45	2.35	0.94
Treatment-emergent resistance				
No	REF			
Yes	46.63	7.76	280.09	**<0.0001**
Known resistance pre-switch				
No	REF			
Yes	<0.0001	<0.0001	<0.0001	**0**
HBV serostatus				
Negative	REF			
Positive	<0.0001	<0.0001	<0.0001	**0**
HCV serostatus				
Negative	REF			
Positive	<0.0001	<0.0001	<0.0001	**0**
Time				
M6	REF			
M12	3.22	0.27	38.23	0.35
M24	<0.0001	<0.0001	<0.0001	**0**
M36	<0.0001	<0.0001	<0.0001	**0**
M42	<0.0001	<0.0001	<0.0001	**0**
3DR*time				
3DR *M6	REF			
3DR *M12	1.23	0.073	20.77	0.88
3DR *M24	<0.0001	<0.0001	<0.0001	**0**
3DR *M36	<0.0001	<0.0001	<0.0001	**0**
3DR *M42	<0.0001	<0.0001	<0.0001	**0**

Abbreviations: OR; odds ratio, CI; confidence interval, GEE; generalised estimating equations, VF; virologic failure, OT; on treatment, RAM; resistance-associated mutation, CRF; circulating recombinant forms, ART; antiretroviral therapy, DTG/3TC; dolutegravir/lamivudine, 3DR; three-drug regimen, M; month.

[a] p values in bold are considered statistically significant.

For Review Only

Table S4. Adjusted ORs, 95% CIs and corresponding p-values from GEE modelling of time to VF; ITT analysis.

**Variable**	**HR**	**2.5 %**	**97.5 %**	**p-value**[a]
ART group				
DTG/3TC	REF			
3DR	2.25	0.64	7.94	0.21
Age	0.96	0.93	0.99	**0.023**
Sex				
Female	REF			
Male	0.34	0.12	0.98	**0.046**
Baseline CD4 count (cells/mL)				
<500	REF			
≥500	0.31	0.12	0.81	**0.017**
RAMs				
No pre-exisiting RAMs	REF			
Pre-existing RAMs	1.10	0.42	2.86	0.84
Missing RAM information	13.55	1.50	122.79	**0.020**

Abbreviations: HR; hazard ratio, CI; confidence interval, GEE; generalised estimating equations, VF; virologic failure, ITT; intent to treat, ART; antiretroviral therapy, DTG/3TC; dolutegravir/lamivudine, 3DR; three-drug regimen, RAM; resistance-associated mutation.

[a] p values in bold are considered statistically significant.

For Review Only

Table S5. Adjusted ORs, 95% CIs and corresponding p-values from GEE modelling of time to VF; OT analysis.

**Variable**	**HR**	**2.5 %**	**97.5 %**	**p-value**
ART group				
DTG/3TC	REF			
3DR	3.13	0.70	14.06	0.14
Age	0.96	0.92	0.99	**0.012**
Sex				
Female	REF			
Male	0.30	0.094	0.97	**0.043**
Baseline CD4 count (cells/mL)				
<500	REF			
≥500	0.37	0.13	1.036	0.058
RAMs				
No pre-exisiting RAMs	REF			
Pre-existing RAMs	1.15	0.41	3.2	0.79
Missing RAM information	16.9	1.80	158.41	**0.013**

Abbreviations: HR; hazard ratio, CI; confidence interval, GEE; generalised estimating equations, VF; virologic failure, OT; on treatment, ART; antiretroviral therapy, DTG/3TC; dolutegravir/lamivudine, 3DR; three-drug regimen, RAM; resistance-associated mutation.

[a] p values in bold are considered statistically significant.

For Review Only

Table S6. PLHIV health and treatment satisfaction rates (A), self-reported adherence (B) and experience of side effects (C) in the InfCareHIV health questionnaire.

A.

How satisfied are you with your physical health?

Response	Pre-switch, N (%)			Post-switch, N (%)		
	**DTG/3TC**	**3DR**	**Total**	**DTG/3TC**	**3DR**	**Total**
Very satisfied	56 (29)	50 (27)	106 (28)	52 (27)	41 (22)	93 (25)
Satisfied	76 (40)	59 (32)	135 (36)	67 (35)	60 (33)	127 (34)
Somewhat satisfied	35 (18)	33 (18)	68 (18)	35 (18)	41 (22)	76 (2)
Somewhat dissatisfied	14 (7)	16 (9)	30 (8)	20 (10)	17 (9)	37 (10)
Dissatisfied	5 (3)	13 (7)	18 (5)	10 (5)	17 (9)	27 (7)
Very dissatisfied	4 (2)	8 (4)	12 (3)	7 (4)	7 (4)	14 (4)
Chose not to answer	0 (0)	3 (2)	3 (1)	0 (0)	0 (0)	0 (0)
Total	190	182	372	191	183	374
How satisfied are you with your mental health?						
Very satisfied	56 (29)	55 (30)	111 (30)	54 (28)	50 (27)	104 (28)
Satisfied	70 (36)	59 (33)	129 (35)	73 (38)	54 (30)	127 (34)
Somewhat satisfied	43 (22)	32 (18)	75 (20)	36 (19)	51 (28)	87 (23)
Somewhat dissatisfied	12 (6)	19 (1)	31 (8)	16 (8)	13 (7)	29 (8)
Dissatisfied	4 (2)	8 (4)	12 (3)	8 (4)	9 (5)	17 (5)
Very dissatisfied	6 (3)	6 (3)	12 (3)	4 (2)	5 (3)	9 (2)
Chose not to answer	1 (1)	2 (1)	3 (1)	0 (0)	1 (1)	1 (0)

For Review Only

Total	192	181	373	191	183	374
How satisfied are you with your sex life?						
Very satisfied	30 (16)	23 (13)	53 (14)	26 (14)	23 (13)	49 (13)
Satisfied	74 (39)	55 (30)	129 (35)	72 (38)	52 (29)	124 (33)
Somewhat satisfied	42 (22)	34 (19)	76 (20)	49 (26)	36 (20)	85 (23)
Somewhat dissatisfied	12 (6)	19 (10)	31 (8)	20 (10)	22 (12)	42 (11)
Dissatisfied	12 (6)	17 (9)	29 (8)	9 (5)	20 (11)	29 (8)
Very dissatisfied	8 (4)	16 (9)	24 (6)	6 (3)	15 (8)	21 (6)
Chose not to answer	13 (7)	18 (10)	31 (8)	10 (5)	14 (8)	24 (6)
Total	191	182	373	192	182	374
How satisfied are you with your care?						
Very satisfied	162 (84)	143 (79)	305 (82)	157 (82)	141 (77)	298 (79)
Satisfied	22 (11)	26 (14)	48 (13)	28 (15)	31 (17)	59 (16)
Somewhat satisfied	5 (3)	8 (4)	13 (3)	6 (3)	7 (4)	13 (3)
Somewhat dissatisfied	0 (0)	1 (0.6)	1 (0.)	0 (0)	0 (0)	0 (0)
Dissatisfied	0 (0)	1 (0.6)	1 (0)	0 (0)	0 (0)	0 (0)
Very dissatisfied	0 (0)	2 (1)	2 (1)	1 (1)	2 (1)	3 (1)
Chose not to answer	3 (2)	1 (0.6)	4 (1)	0 (0)	2 (1)	2 (1)
Total	192	182	374	192	183	375
B.<br>*Do you take your medication?*						

For Review Only

**Response**	**Pre-switch, N (%)**			**Post-switch, N (%)**		
	**DTG/3TC**	**3DR**	**Total**	**DTG/3TC**	**3DR**	**Total**
Yes	246 (99)	268 (100)	514 (99)	249 (100)	267 (99)	516 (100)
No	0 (0)	1 (0)	1 (0)	0 (0)	0 (0)	0 (0)
Chose not to answer	2 (1)	0 (0)	2 (0)	0 (0)	2 (1)	2 (0)
Total	248	269	517	249	269	518
How many doses did you miss last week?						
0 doses	216 (89)	230 (87)	446 (88)	229 (93)	232 (87)	461 (90)
1–2 doses	24 (10)	32 (12)	56 (11)	16 (7)	31 (12)	47 (9)
3 or more	2 (1)	0 (0)	2 (0)	0 (0)	2 (1)	2 (0.5)
Chose not to answer	2 (1)	2 (1)	4 (1)	1 (0)	1 (0)	2 (0.5)
Total	244	264	508	246	266	512
C.<br>*Are you currently experiencing any side effects?*						
**Response**	**Pre-switch, N (%)**			**Post-switch, N (%)**		
	**DTG/3TC**	**3DR**	**Total**	**DTG/3TC**	**3DR**	**Total**
Yes	23 (9)	37 (14)	60 (12)	19 (8)	30 (11)	49 (10)
No	216 (89)	222 (84)	438 (86)	221 (9)	230 (87)	451 (89)
Chose not to answer	5 (2)	5 (2)	10 (2)	6 (2)	3 (1)	9 (2)
Total	244	264	508	246	263	509
To what extent are you bothered by side effects?						

For Review Only

Very bothered	2 (2)	5 (3)	7 (3)	2 (2)	4 (3)	6 (2)
Bothered	9 (7)	10 (7)	19 (7)	4 (3)	7 (5)	11 (4)
Somewhat bothered	8 (6)	13 (9)	21 (8)	9 (7)	11 (7)	20 (7)
Not that bothered	13 (10)	23 (16)	36 (12)	14 (11)	19 (13)	33 (12)
Not at all bothered	68 (54)	69 (47)	137 (50)	66 (53)	72 (49)	138 (51)
Chose not to answer	27 (21)	27 (18)	54 (20)	29 (23)	35 (24)	64 (24)
Total	127	147	274	124	148	272

Abbreviations: DTG/3TC; dolutegravir/lamivudine, 3DR; three-drug regimen.

For Review Only
